# Supplementary material for: Chromothripsis during telomere crisis is independent of NHEJ, and consistent with a replicative origin
Source: Genome Res. 2019 May;29(5):737–49. doi: 10.1101/gr.240705.118 (PMC6499312; doi:10.1101/gr.240705.118)
Supplement: Supplemental Material [file supp_gr.240705.118_Supplemental_file_1.zip › contigs/annotated_contigs/DB78/contig.2.DB78_length_557_mean_cov_8.8868940754.docx]

**DB78_length_557_mean_cov_8.8868940754**

```
TTTTCATTTCTGTAAAGTTTTCCATGATGCAGATCCTGACCTCAGCATTTTACCTGTATTAACCCATTTAATCTAACAATCATTCTATA
 >chr2:19694496-19694779 + E=1e-158
GGTAGGTGCTATGACTACATTTTTATAAATGAGGAAACTGAGGTCAACAAGAGCAATTGAGAGCTCCTTCATAACAGAGCCAGAGAATG

AAACCTAGGCAGTCTTATTACACTGATGGGCCAGAAATATCCACAATGCCAGAACCACGTAATGTTGCAGCTGGAGGACAACACAGAAA

AAATCTAGTAAAT|CTC|CATTCACTCTTTTGCTGCTTTTTTTAATTTTTAATTTTTTTATTTCAATAGGTTTTTGGGGAACAGGTTGT
              >chr2:19710882-19711160 + E=8e-148
GTTTGGTTACATGAATAAGTTCTTGAGTGGTGATTTCTGATATTTTGATGCACCCATCACCTGAGTAGTATATACTGTACCCAATGTGT

AGTCTTTTATCCCTTGCCACCCCCCACCCTTTCCCCTAAGTCCCCAAAGTACAATGTATCATTCTTATGCCTTTGTGTCCTCATGGCTT

AGCTCCCACATATGCATGAGAACGT
```
